# Use of ChatGPT in Urology and its Relevance in Clinical Practice: Is it useful?

**DOI:** 10.1590/S1677-5538.IBJU.2023.0570

**Published:** 2024-03-18

**Authors:** Antonio Vitor Nascimento Martinelli Braga, Noel Charlles Nunes, Emanoel Nascimento Santos, Maria Luiza Veiga, Ana Aparecida Nascimento Martinelli Braga, Glicia Estevam de Abreu, José de Bessa, Luis Henrique Braga, Andrew J. Kirsch, Ubirajara Barroso

**Affiliations:** 1 Escola Bahiana de Medicina e Saúde Pública Centro de Distúrbios Urinários Infantis Salvador BA Brasil Centro de Distúrbios Urinários Infantis (CEDIMI), Escola Bahiana de Medicina e Saúde Pública, Salvador, BA, Brasil;; 2 Universidade Estadual de Feira de Santana Faculdade de Medicina Feira de Santana BA Brasil Faculdade de Medicina, Universidade Estadual de Feira de Santana, Feira de Santana, BA, Brasil;; 3 McMaster University Hamilton Ontario Canada McMaster University, Hamilton, Ontario, Canada;; 4 Children's Healthcare of Atlanta and Emory University School of Medicine Pediatric Urology Atlanta GA United States Pediatric Urology, Children's Healthcare of Atlanta and Emory University School of Medicine, Atlanta, GA, United States

**Keywords:** Urology, Vesico-Ureteral Reflux, Cakut [Supplementary Concept]

## Abstract

**Purpouse:**

One of the many artificial intelligence based tools that has gained popularity is the Chat-Generative Pre-Trained Transformer (ChatGPT). Due to its popularity, incorrect information provided by ChatGPT will have an impact on patient misinformation. Furthermore, it may cause misconduct as ChatGPT can mislead physicians on the decision-making pathway. Therefore, the aim of this study is to evaluate the accuracy and reproducibility of ChatGPT answers regarding urological diagnoses.

**Materials and Methods:**

ChatGPT 3.5 version was used. The questions asked for the program involved Primary Megaureter (pMU), Enuresis and Vesicoureteral Reflux (VUR). There were three queries for each topic. The queries were inserted twice, and both responses were recorded to examine the reproducibility of ChatGPT's answers. Afterwards, both answers were combined. Finally, those rwere evaluated qualitatively by a board of three specialists. A descriptive analysis was performed.

**Results and Conclusion:**

ChatGPT simulated general knowledge on the researched topics. Regarding Enuresis, the provided definition was partially correct, as the generic response allowed for misinterpretation. For VUR, the response was considered appropriate. For pMU it was partially correct, lacking essential aspects of its definition such as the diameter of the dilatation of the ureter. Unnecessary exams were suggested, for Enuresis and pMU. Regarding the treatment of the conditions mentioned, it specified treatments for Enuresis that are ineffective, such as bladder training. Therefore, ChatGPT responses present a combination of accurate information, but also incomplete, ambiguous and, occasionally, misleading details.

## INTRODUCTION

One of the many AI-based tools that has gained popularity is the Chat-Generative Pre-Trained Transformer (ChatGPT) (1, 2).

Today, ChatGPT is used thoroughly, as a “Search Engine”, by patients and physicians, seeking adjuvant information and knowledge about their disease (2–4). However, due to its popularity, and its massive media coverage, incorrect and misleading information provided by ChatGPT will have a profound impact, leading patients and physicians to misinformation (3, 4). Furthermore, it may cause misdiagnosis and mistreatment as ChatGPT can lead the physician to the wrong path on the decision-making chart (3–6).

Consequently, it is essential to evaluate and assess its accuracy and consistency regarding urological queries. The aim of this study is to evaluate and investigate the reliability of ChatGPT 3.5 in terms of concept description, as well as usefulness for decision-making in clinical practice regarding urology.

## METHODS

ChatGPT 3.5 version from March 14 was used. The questions asked for the LLM program involved three urological conditions, Primary Megaureter (pMU), Enuresis and Vesicoureteral Reflux (VUR), that were chosen due to a certain ambiguity regarding its diagnosis and treatment.

Regarding the number of questions prompt to ChatGPT, there were 3 queries for each of the topics mentioned above adding up to 9. All the questions provided to ChatGPT are summarized on Table-1.

**Table 1:** Questions to ChatGPT according to pathology and its level of response.

PATHOLOGY	QUESTION	CHATGPT'S TYPE OF ANSWER
ENURESIS	What is the definition of Enuresis?	Type 2
	How to diagnose Enuresis in a two-page long answer?	Type 1
	How to treat Enuresis in a two-page long answer?	Type 1
VESICOURETERAL REFLUX	What is the definition of Vesicoureteral reflux?	Type 3
	What are the indications for endoscopic injection treatment for Vesicoureteral reflux in a two-page long answer?	Type 1
	What are the results for endoscopic injection treatment for Vesicoureteral reflux, in a two-page long answer?	Type 2
PRIMARY MEGAURETER	What is the definition of Primary Megaureter?	Type 2
	How to diagnose Primary Megaureter in a two-page long answer?	Type 1
	How to treat Primary Megaureter in a two-page long answer?	Type 1

Subtitle:

Type 1 - Misleading/Negative Impact to Care.

Type 2 - Helpful to lay person but lacking in substance.

Type 3 - Useful information for patients and treating healthcare providers.

Each question was entered as a separate, independent prompt using the “New Chat” function and an anonymous window to minimize bias. The suffix “in a two-page long answer” was used to assure that ChatGPT would provide a similar length answer to every query. The queries were inserted into ChatGPT twice, at the same time, and both responses were recorded to examine the reproducibility of ChatGPT's answers.

Afterwards, both questions were combined, forming a single answer. Subsequently, those responses were evaluated qualitatively by a board of three specialists, considered thought leaders in the field (AK, LB and UB), each providing a report regarding the accuracy of ChatGPT.

Descriptive analyses were performed. ChatGPT answers were categorized in three types, according to its relevance in clinical practice and patient's information (Table-1).

A table (Table-1) with ChatGPT's type of answer was generated.

## RESULTS

The information contained in the ChatGPT responses was analyzed by urologists who are experts in the field and have extensive clinical experience in the respective pathologies.

## ENURESIS

The ChatGPT defines enuresis as “medical term that refers to the involuntary discharge of urine, usually during sleep, in a person who is beyond the age of toilet training. It is commonly known as bedwetting”. However, by definition, enuresis always and not usually occurs during sleep. The usage of the term “usually” in the previous explanation introduces ambiguity and imprecision.

When ChatGPT is asked about the diagnosis of enuresis, it replies that it can be done through “clinical history, physical examination and complementary tests”. Regarding the clinical history, the specialist highlighted that crucial aspects were omitted during the anamnesis, including the evaluation of daytime urinary symptoms, and psychological abnormalities, which are essential for making an accurate diagnosis.

The evaluation of the lumbosacral region, which is essential for patients with urinary symptoms, should be highlighted, as it was not mentioned by ChatGPT. Furthermore, the possible presence of bladder prolapse or enlarged prostate should not be included.

We found the following errors and inconsistencies regarding complementary tests: in relation to ultrasonography, the evaluation of the post void residual is not mentioned and there is no indication of performing a urodynamic study for the diagnosis of enuresis, as well as blood tests. Magnetic resonance imaging would only be indicated in very particular situations. In addition, there is no citation of the voiding diary and nocturnal calendar, which are essential tools.

ChatGPT's answer in regard to treatment contains inaccurate information regarding the effectiveness of bladder training for treating enuresis. The statement suggesting that bladder training involves encouraging the patient to hold urine for progressively longer periods and gradually increasing the time between urinations is incorrect.

Regarding treatment, we found some inconsistency and some missed information. Bladder training does not work for monosymptomatic enuresis. It does not mention the dose, duration of treatment and the main side effect of the medications, that is, hyponatremia for desmopressin and cardiotoxicity for imipramine; nothing is said about the success rate of the alarm. ChatGPT provided satisfactory comments on urotherapy, however, it falls short in terms of medication guidance and incomplete information about the alarm method. The lack of details on medication usage, main side effects, success rate, and duration of use hampers the ability to make informed decisions based on the description of enuresis treatment provided by ChatGPT.

## VESICOURETERAL REFLUX

We have no comment on the definition of this condition provided by ChatGPT, indicating that no inconsistencies were found in the answer.

On the other hand, the response regarding the indication of endoscopic treatment of VUR presented some incomplete and even wrong information. Deflux is FDA approved for grades II-IV and also for duplex ureters that are not mentioned in the text.

For patients with high surgical risk, ChatGPT says that endoscopic injection therapy may be indicated for patients who are at high risk for surgical complications. This information is false, as the procedures are performed under anesthesia in an operating room. Thus, high surgical risk patients are at the same anesthetic risk.

ChatGPT also mentions the treatment of reflux associated with voiding dysfunction. This statement is false because endoscopic injection does not correct lower urinary tract disfunction *per se*. It can be used in this situation, but it does not correct the filling and emptying changes and may result in a lower success rate than children without lower urinary tract disfunction.

When inquired about the outcomes of endoscopic injection for the treatment of reflux, ChatGPT provides a list of points, as outlined below.

Initially, the answer generated talks about preventing recurrent urinary tract infections. It was not considered the experience with more than 5 years of follow-up is 94% of clinical success (7).

Regarding the improvement of kidney function, we found in the answer the explanation that “VUR can cause damage and scarring in the kidneys”. Preservation of kidney function is difficult to define, as most grades 1-3 have normal function, so they achieve preserved kidney function. Furthermore, when high success rates are cited in the excerpt, the long-term data is missing.

The answer also includes information about low complication rates, and ChatGPT mentions the possibility of ureteral perforation. We are not aware about this specific complication.

## PRIMARY MEGAURETER

Concerning the definition of this condition, ChatGPT has been created in lay-term and imprecise language, avoiding using medical terms such as “ureter”. Furthermore, any definition of Primary Megaureter (pMU) must include the dilatation of the ureter, which is exactly what defines this condition, a discussion that ChatGPT does not mention.

In addition, ChatGPT doesn't mention any cutoff point of ureteral dilatation, which is greater than 7mm measured behind the bladder on ultrasonographic transverse view. ChatGPT also continuous to be unspecific as it does not classify pMU in refluxing, obstructive or refluxing obstructive megaureter. The AI platform mentions that megaureter is typically diagnosed in childhood, however most cases are diagnosed antenatally.

Regarding the diagnosis of pMU ChatGPT level of accuracy is low, with generic and incorrect statements. ChatGPT doesn't mention the subtypes that may need a Lasix renal scan (MAG3 or DTPA), a VCUG to rule out VUR or even other tests such as MRU. However, most cases are diagnosed prenatally, therefore physical exam is not required. It also says that the physician needs to palpate the bladder, however, once the problem occurs above the bladder level, bladder is not distended in pMU, showing that ChatGPT gave an incorrect statement.

ChatGPT also says that imaging studies, such as ultrasound is needed; however, its answer is very simplistic, doesn't mentioning the minimal diameter of the ureter, where it should be measured, the grades of hydronephrosis and other aspects that impacts the medical decision. ChatGPT also recommends doing Voiding cystourethrogram (VCUG), once, according to ChatGPT, it can identify VUR. However, VUR is not a complication of pMU, it is considered one of the three types of megaureter, showing a mistake. Renal scan is mentioned by ChatGPT correctly, but it doesn't mention its major importance: provide information about the drainage of the urinary system which is essential in cases of pMU. ChatGPT also says that urine culture is necessary, but doesn't show the indications of the exam, that's only needed if the child develops a UTI, not being necessary for every patient with pMU. It implied that most patients should have a urine test done as part of the diagnostic work up, which is incorrect. Tests such as renal functional such as glomerular filtration rate (GFR) are also mentioned; however, most children will not need these tests, as the condition is unilateral. The major mistake of ChatGPT is that it doesn't mention the prenatal diagnosis, once there is no way to suspect that a baby has pMU, and most patients are born asymptomatic.

About the treatment options, ChatGPT says correct sentences, such as, it depends on the severity of the condition and any associated complications, the age and overall health of the patients, and that the goals of treatment are to prevent infections, protect kidney function and relieve symptoms if present. However, it doesn't mention the watchful waiting on antibiotics, once evidence has shown that there is a role for antibiotic prophylaxis in patients with pMU, reducing the rate of UTI.

Regarding endoscopic surgery, ChatGPT states that it corrects the dilatation of the ureter, which is not correct. It is the opposite once it corrects the narrow distal segment of the ureter by stretching it with a balloon or a stent. ChatGPT also states that laparoscopic surgery results in less pain and faster recovery rates; however, this has not been shown in cases of tapered ureteral reimplantation. No comparative studies have been done with those pathologies, not been possible to state a sentence like that. It also says that nephrectomy is indicated in some cases that the affected kidney isn't functioning properly or causing complications; however, it is extremely unlikely to have a kidney completely damaged due to pMU.

## DISCUSSION

According to Evidence Based Medicine medical decisions should be based on the latest medical research evidence to provide the most appropriated treatment and diagnosis plan for the patient (6). AI has the potential to bring several benefits in medical knowledge, such as improving clinical decision-making and contributing to education, once by making direct questions, ChatGPT gives almost instant answers based on high level evidence.

However, there are concerns related to excessive confidence in technology and ethical issues in its use. At this time, ChatGPT lacks genuine clinical experience and judgment, and may provide wrong information. Urology is a field of medicine with complex pathologies that doesn't necessarily have direct answers and unique diagnosis or types of treatment, making the clinician's experience indispensable. ChatGPT performs well in less complex questions; however, its performance decreases as the complexity of medical decisions increases. It demonstrates knowledge equivalent to a third-year medical student, as shown on Aidan Gilson et al study, based on its performance in United States Medical Licensing Examination® responses (8).

Numerous cardiology and oncology approaches have demonstrated the utility of AI, particularly in identifying and classifying disease phenotypes and improving predictive outcome models by incorporating unstructured data (9–12). Using AI to identify inhaler techniques in electronic health records for asthma care, a study suggests it may be possible to eliminate the expensive manual chart review required for guideline-conformant documentation in asthma care by employing a machine learning strategy (9). However, to the best of our knowledge, no study has evaluated the impact of ChatGPT on medical decision-making in Urology, regarding those three specific diagnoses. This could lead to a greater efficacy of medical information for the patient, a higher rate of treatment adherence, and a reduction in treatment costs, as well as the secondary effects of incorrectly treating prevalent diseases, such as the ones we analyzed.

In our study, we analyzed the quality of the information provided by ChatGPT in responses on relevant topics in urology. ChatGPT provided wrong answers to important topics, such as, the definition of enuresis, which was partially correct. It gave generic responses that did not align with reality. Concerning the evaluation and diagnosis, ChatGPT omitted crucial aspects of anamnesis, which is an essential part of clinical evaluation and defines decision-making.

After analyzing the response regarding enuresis, we identified unnecessary diagnostic exams being suggested, including urodynamic studies and magnetic resonance imaging.

Regarding the endoscopic treatment of vesicoureteral reflux, we had a response that could confuse the reader regarding the method's efficacy. This is due to the lack of citation of results found in relevant published studies on the subject, as well as suggesting outcomes that are not realistic, such as the resolution of urinary symptoms (13–15). This error in treatment is repeated in the response about enuresis, which mentions ineffective treatments such as bladder training.

Our findings concur with those of Katharina Jeblick et al., who examined the use of ChatGPT in radiology reports. They found both accurate and incorrect statements, which were categorized into four error categories: misinterpretation of medical terms, imprecise language, hallucination, strange language, and grammatical errors (16). While we did find accurate information, given ChatGPT's broad background, the language models employed lack specialized medical understanding and knowledge, resulting in imprecise responses that occasionally contain phrases from previous interactions (17, 18).

Therefore, ChatGPT is a tool that can facilitate public access to information. However, these technologies must be upgraded to enhance the comprehension of medical questions and facilitate clinical decision-making by providing more specific answers and fewer generic texts.

## CONCLUSIONS

ChatGPT responses contain a mosaic of accurate and pertinent information; however, the majority of its responses contain broad, insufficient, and misleading information. In the face of the experts’ feedback and evaluations, it is not recommended to base clinical and therapeutic decisions solely on ChatGPT's knowledge. It is important to disseminate this information to non-expert professionals and patients, given that ChatGPT has received significant media attention and is widely accessible to the public. In this way, we seek to protect users from harm.
